# Acute Appendicitis Masquerading Distal Intestinal Obstruction Syndrome in Adult Cystic Fibrosis

**DOI:** 10.1155/2018/8475060

**Published:** 2018-09-26

**Authors:** Sushant M. Nanavati, Hiren Patel, Gabriel Melki, Vinod Kumar, Edward Milman, Patrick Michael, Ariy Volfson

**Affiliations:** ^1^Department of Internal Medicine, St. Joseph's University Medical Center-New York Medical College, USA; ^2^Department of Gastroenterology, St. Joseph's University Medical Center-New York Medical College, USA; ^3^Department of Radiology, St. Joseph's University Medical Center-New York Medical College, USA

## Abstract

Overshadowed by Sino-pulmonary infections, Cystic Fibrosis (CF) commonly affects gastrointestinal organs because of secretory and motility dysfunction. Infrequently, these changes result in Distal Intestinal Obstruction Syndrome (DIOS), an increasingly diagnosed gastrointestinal entity in adult Cystic Fibrosis patients. We present a case 22-year-old male who presented to our hospital with right lower quadrant abdominal pain with suspicion of acute appendicitis and was subsequently diagnosed as DIOS. Our case highlights the importance of DIOS as one of the differential diagnosis of right lower quadrant abdominal pain in a patient with a CF, especially for physicians working at community hospitals which may not have a Cystic Fibrosis care program available.

## 1. Introduction

Cystic Fibrosis (CF) is a genetic disease affecting multiple organs. With the advancement in the management of CF patients, patients now often survive well into adulthood [1]. Improved life expectancy among adult CF patients has given rise to extrapulmonary, notably gastrointestinal, manifestations which were not previously encountered. Distal Intestinal Obstruction Syndrome (DIOS) continues to be a rising complication in adult CF patients presenting with acute abdominal pain mimicking an acute abdominal emergency. 

## 2. Case Report

**Figure 1:**
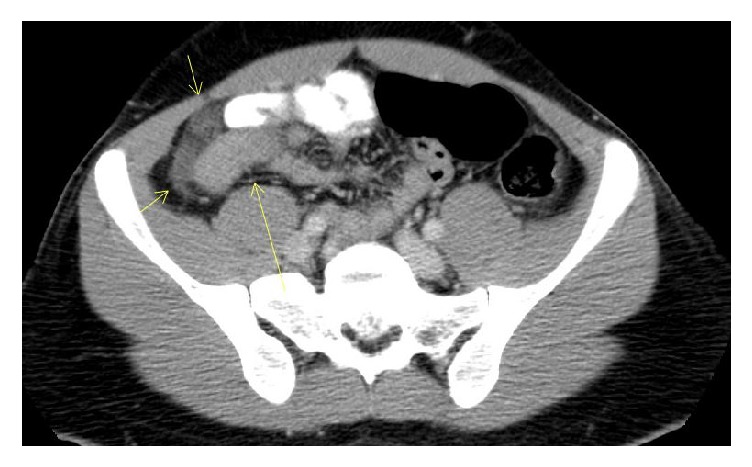
Axial abdominal CAT scan depicting thickening around the terminal ileum and colon (Yellow arrows) along with extraluminal fluid and reactive lymph nodes.

**Figure 2:**
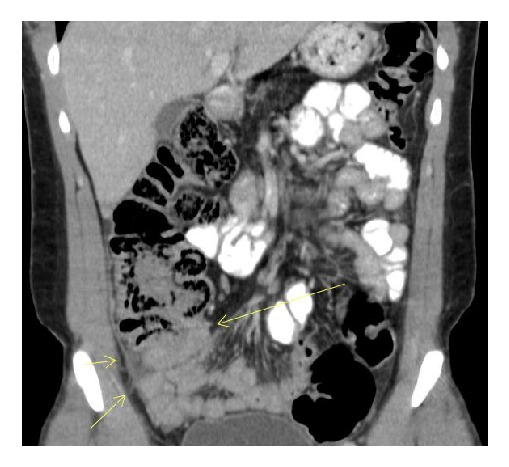
Coronal view with thickening of ileum with distended appendix (yellow arrows).

A 22-year-old Turkish-origin male with a past medical history of Cystic Fibrosis presented with a one-day history of right lower quadrant abdominal pain. He described sharp periumbilical pain that continued to worsen which then shifted to right lower quadrant abdomen. Prior to the onset of the abdominal pain, he reported experiencing nausea and anorexia for three days. His last bowel movement was two days prior to admission. The patient was diagnosed with Cystic Fibrosis at the age of four and his disease progressed to exocrine pancreas insufficiency, which was being treated with pancreatic enzymes. Upon reviewing the patient's past history, it was noted that he had several episodes of pneumonia for which he was appropriately treated with antibiotics; notably, no history of constipation or recurrent abdominal discomfort was reported prior. At home, the patient was prescribed Albuterol inhaler as needed, Dornase Alfa inhaler, Aztreonam lysine nebulization, Azithromycin 500 mg three times a week, Lansoprazole, Lumacaftor-ivacaftor twice a day, Lipase-protease-amylase capsule three times a day, and a multivitamin capsule once a day. On abdominal exam, he had diminished bowel sounds and tenderness on right lower quadrant with equivocal rebound tenderness. Laboratory analysis showed leukocytosis (WBC 13.0 mm/K3, Neutrophils 62%) with a normal differential. He had no electrolyte imbalances. Computed Tomography (CT) of the Abdomen revealed thickening and edema around the terminal ileum, a colon with inflammatory changes, free fluid in the right paracolic gutter adjacent to the cecum, an appendix measuring 5.3 × 4.6 mm, and reactive lymph nodes (Figures 1 and 2). Due to extraluminal fluid and cecal wall edema with inflammation, early acute appendicitis could not be excluded as a diagnosis. Surgical intervention was performed which revealed a ruptured microperforation of a cecal diverticulum and a distended appendix in chronic adhesions for which he required an appendectomy and partial cecectomy with intact ileocecal valve (IC valve). Postoperatively, he was diagnosed with DIOS and subsequently started on Polyethylene Glycol. The patient made an unremarkable recovery and was discharged home to be followed up in the outpatient clinic without any recurrence of any symptoms.

## 3. Discussion

Distal Intestinal Obstruction Syndrome (DIOS) was called a Meconium Ileus-equivalent in the past, described by the collection of viscid fecal material within the lumen combined with sticky mucoid intestinal content adherent to the intestinal wall of the terminal ileum and cecum [1]. Perez-Aguilar et al. reported a prevalence of 19.5% (mean age 20.6 years) among 46 CF patients in a retrospective analysis, while Dray et al. conducted a cross-sectional study reporting a 15.8% (mean age 28.9 years) prevalence in 171 CF patients [2, 3]. Though there continues to be a limited assessment of the prevalence of DIOS in adult CF, DIOS is considered common among adults compared to children due to increased disease progression.

Defective intestinal chloride and water secretions into the gut, luminal acidity, and loss of bile salt all contribute to the development of DIOS [1]. These patients characteristically present with right lower quadrant pain, nausea, abdominal distension, and failure to pass stools or flatus [1, 3]. In some patients, a palpable right lower quadrant mass can be appreciated that may be confirmed on abdominal X-ray [1]. Though abdominal X-rays are recommended to aid in the diagnosis of DIOS, they are inadequate in differentiating ileus from other causes of abdominal pathologies that may present in Cystic Fibrosis patients [4]. Due to the proximity of the anatomical locations, as well as the overlapping clinical presentations, appendicitis and intussusception may mimic DIOS which further leads to diagnostic uncertainty. Overlap of several intra-abdominal pathologies in CF increases the risk of misdiagnosis, especially with acute appendicitis, as these patient's underlying pathologies may be masked with antibiotics prescribed for pulmonary infections [5, 6].

Osmotic laxatives are the cornerstone of bowel regimens for the treatment of DIOS. The most commonly prescribed is Polyethylene Glycol (PEG) administered at a dose of 20-40 ml/Kg/H, up to a maximum of 1 L/h for a total of 8 hours, achieving fecal effluent consisting of clear fluid along with resolution of abdominal pain and constipation [1, 6]. If the diagnosis remains unclear and thus requires surgical intervention, ileocecal valve resection should be considered to prevent the recurrence of intestinal obstruction sequalae and growth, especially in adolescents [7].

With the increase in immigration of foreigners through America, inner-city and community hospitals may not be equipped with a Cystic Fibrosis care center; nor may these hospitals have programs in provision with expertise available to other clinicians involved in patient care. Therefore, our case highlights the importance of considering DIOS as a differential diagnosis in a patient presenting with the history of CF and complaints of abdominal pain and constipation.
